# Motivational foci and asthma medication tactics directed towards a functional day

**DOI:** 10.1186/1471-2458-11-809

**Published:** 2011-10-17

**Authors:** Malin Axelsson, Jan Lötvall, Jesper Lundgren, Eva Brink

**Affiliations:** 1 Department of Nursing, Health and Culture, University West, SE-461 86, Trollhättan, Sweden; 2 Krefting Research Centre, Institute of Medicine, Internal Medicine, Sahlgrenska Academy, University of Gothenburg, SE-405 30, Gothenburg, Sweden; 3 Department of Psychology, University of Gothenburg, SE-405 30, Gothenburg, Sweden; 4 Institute of Health and Care Sciences, Sahlgrenska Academy, University of Gothenburg, SE-405 30 Gothenburg, Sweden

## Abstract

**Background:**

There appears to be an obvious gap between a medical and patient adherence perspective. Deviating from a medication prescription could be regarded as fairly irrational, but with respect to patients' goals and/or concerns it could be seen as understandable. Thus, the aim was to elucidate adherence reasoning in relation to asthma medication.

**Methods:**

This was a qualitative study; data collection and analysis procedures were conducted according to Grounded Theory methodology. Eighteen persons, aged 22 with asthma and regular asthma medication treatment, were interviewed.

**Results:**

The emerged theoretical model illustrated that adherence to asthma medication was motivated by three foci, all directed towards a desired outcome in terms of *a functional day as desired by the patient*. A *promotive focus *was associated with the ambition to achieve a positive asthma outcome by being adherent either to the received prescription or to a self-adjusted dosage. A *preventive focus *was intended to ensure avoidance of a negative asthma outcome either by sticking to the prescription or by preventively overusing the medication. A *permissive focus *was associated with unstructured adherence behaviour in which medication intake was primarily triggered by asthma symptoms.

**Conclusions:**

As all participants had consciously adopted functioning medication tactics that directed them towards the desired goal of a functional day. In an effort to bridge the gap between a patient- and a medical adherence perspective, patients need support in defining their desired functionality and guidance in developing a person-based medication tactic.

## Background

A novel trend direction in health care has been established in recent years. It is seen in the transition from disease-oriented care [1] to a more patient-centred approach [2], which is intended to result in a shared decision about treatment between the patient and the caregiver [1]. It has been argued that a patient-centred approach is crucial for encouraging adherence to treatment [3]. With reference to asthma medication treatment, several studies have reported figures showing lower adherence than prescribed [4-8]. From a medical perspective, deviating from a medication prescription could seem rather irrational, because it could constitute the missing link between a prescribed treatment and an efficacious outcome [9]. Inadequate adherence to asthma medication can be traced to poor asthma outcome [10,11] and more frequent health-care seeking [12], with increasing health-care costs as a consequence [13].

From a patient perspective, deviating from a prescribed treatment could seem rational; for instance, regular medication intake is incongruent with denial of an asthma diagnosis or with the belief that asthma is an acute illness [14]. Patients' perception of asthma seems to influence adherence behaviour, as those who do not experience any symptoms [15] or start to feel better when medication is initiated tend to deliberately interrupt the medication treatment [16]. By contrast, patients who perceive their asthma as severe [15] or subjectively experience symptom relief [16] are more likely to adhere to prescribed asthma medication [15,16]. The conviction that the best way to prevent asthma symptoms from occurring is to combat asthma triggers, such as infection or stress, tends to be associated with a preference for alternative treatment methods [17]. Beliefs about medication are known to play a significant role for adherence behaviour. Patients who regard the asthma medication as a necessity for their health are more likely to follow their prescriptions [15,18,19], unlike those who are concerned with side effects or are afraid of becoming dependent [18,19].

From a psychological perspective, there are indications that personality traits influence medication adherence [20-26]. From an integrative personality perspective, personality traits in conjunction with human nature, culture, life narratives and characteristic adaptation need to be considered if we are to understand the individual as a whole. Characteristic adaptation refers to how individuals tackle everyday life and speaks to the motives, goals, plans and so forth that can be explained by cognitive-motivational strategies [27]. As such, Higgins's theory of self-regulatory focus as a motivational principle [28] explains individual differences in goal-directed behaviour in terms of two distinct systems: promotion and prevention. Although both systems are directed towards the same goal, they operate somewhat differently. The promotion system focuses on the achievement of a positive outcome, while the prevention system focuses on the avoidance of a negative outcome [28].

The present study was conducted to gain a broader and deeper understanding of adherence behaviour in young adults by studying it in light of personal characteristic adaptation. Thus, the aim was to elucidate adherence reasoning in relation to asthma medication.

## Methods

To get a broader picture of adherence behaviour, a qualitative design was used in which data were derived from interviews. The sampling and analysis procedures were carried out in accordance with Grounded Theory (GT), as described by Strauss and Corbin [29]. Epistemologically, this version of GT is situated between the classic version, which is more positivistic in nature, and the constructivist or post-modern version [30].

### Study participants

The participants (n = 18) were sampled from an epidemiological study of young adults with asthma born between 1985 and 1987, who had been prescribed regular asthma medication and who had completed an adherence questionnaire [20]. A purposive sampling procedure [31] was applied, resulting in recruitment of participants with various reported adherence scores [20]. Characteristics of the participants are shown in Table 1. The eligible participants were invited to take part by telephone. Both verbal and written information were provided, with an emphasis on voluntary participation.

**Table 1:** Characteristics of the participants

	n
**Age**	

22 (+/- 1 year)	18

**Sex**	

Women	13

Men	5

**Asthma onset**	

1985-1989	10

1992-1996	6

2000	1

Missing data	1

**Medication**	

Combined+SABA*	13

ICS+SABA^#^	5

**Education level**	

Grammar/high school	7

University	11

**Monthly income in SEK**	

< 10.000	10

10.000-20.000	6

20.000-30.000	2

**Smoking habits**	

Occasional smoker	1

Non-smoker	17

**Emergency visit, the last 12 months**	

Yes	2

No	16

**Oral corticosteroids, the last 12 months**	

Yes	2

No	16

**Occupation**	

Employee	6

Student	10

Other	2

* = Combined inhaler (Single inhaler combining corticosteriods and long-acting beta-2 agonist (LABA))

^# ^= Inhaled corticosteriods (ICS), Short-acting beta-2 agonist (SABA)

### Data collection

Data were collected through interviews, during which the participants were asked to speak as freely as possible about their adherence behaviour. Prior to the interview, each participant's informed consent was obtained. The interviews lasted on average 45 minutes, were recorded digitally and transcribed verbatim.

### Rigour

An important consideration in GT is the balance between theoretical sensitivity [29] and the interaction between the researcher and the data, which is termed reflexivity [32,33]. This delicate matter was dealt with by maintaining constant responsiveness throughout the data collection and analysis. Ideas arising from the analysis were reconfirmed during subsequent collection of new data. Non-confirmed ideas could thereby be discarded. This handling of the data is in line with what in the GT tradition is referred to as constant comparison [29]. In addition, comparisons with literature on adherence and health behaviour were conducted to stimulate thinking during the examination of properties and dimensions in the current data. To maintain a sceptical stance, formulated concepts and categories were validated in subsequent interviews. Occasionally, the data were kept at a distance for a period of time, which allowed the researchers to return to them with a critical eye, ready to judge whether the provisional theory still fitted the data [29].

### Analysis

**Figure 1:**
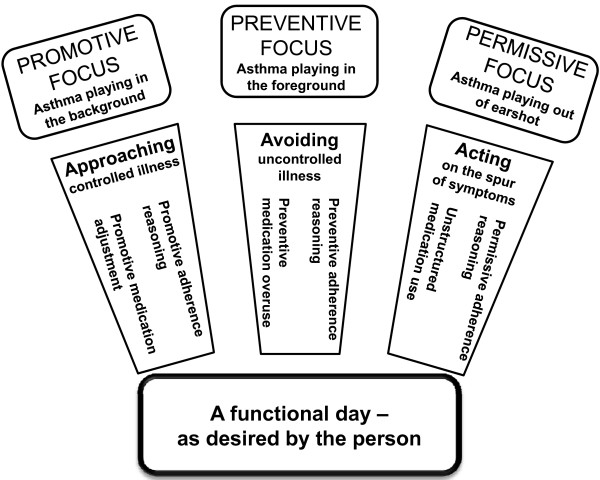
**A theoretical model illustrating adherence behaviour in young adults with asthma**.

The sampling procedure was carried out hand-in-hand with the analysis of interview data, in accordance with GT [29]. The computer-based program NVivo was used to handle and organize the data [34]. The initial interviews began in a rather unprejudiced fashion with an open-ended question about medication intake that was followed by probing questions. This was a way for the researchers to keep their minds open to the diversity of incidents and to minimize the risk of forcing the interview in a given direction. Gradually, the interviews became more systematic so as to enable comparisons with the ideas emerging from the analysis. The interviewer still strived for a flexible stance to permit new events and directions in the interviews. Directly after an interview, a memo was written in order to document the interviewer's instant impression of the collected data. The interviews were transcribed verbatim as soon as possible, and the analysis followed. The analysis procedure was a constant comparison of data as regards similarities and differences, with a focus on ongoing processes that started with a microanalysis close to the data. During this phase, the data were examined by asking "How? What? Why?" questions. This part of the analysis generated codes that, depending on their content, were organized into preliminary categories and labelled based on contents of meaning. As an example, several codes contained different motives for the young adult's adherence behaviour such as *"hoping for well-being" *and *"avoiding a risk"*. These formed a preliminary category named *"having a motive for adherence behaviour"*. During the ensuing interviews, the preliminary categories were tested as regards dimensions and properties to enable the building of more solid categories. The subsequent analysis continued by selecting the categories of significance in relation to the aim, and the properties of these categories were defined. This so-called focused coding disclosed an obvious variation in adherence motives that deserved a proper explanation. To stimulate the creative thinking needed during this phase, appropriate literature was used as a cache for inspiration [29]. By coincidence, the researchers found [35] Higgins's theory of self-regulatory focus as a motivational principle [28], which comprises two systems: prevention and promotion foci. These were the parts in the category thus far labelled *"having a motive for adherence behaviour" *that had been overlooked and lacking. At this point, three more refined categories labelled *"promotive focus" *and *"preventive focus" *were developed. The third category, *"permissive focus"*, could perhaps be seen as an extension of Higgins's theory [28], because it was based on data that did not fit into that theory. The following axial coding produced subcategories such as *"approaching illness control"*. Early in the analysis process, the researchers got the impression that *"illness control" *seemed to be fundamental to adherence behaviour. The theoretical sampling was then concentrated on discovering aspects of this concept. The interview questions were more directed at defining and specifying the importance of experienced illness control in relation to adherence. Already collected data were returned to in order to see if this association could be observed, and if so, to reorganize the data. Concurrent with the sampling and the writing of analysis memos, diagrams of relationships between categories were drawn. Eventually, a core category emerged - *"A functional day as desired by the person" - *to which the other categories could be related. A theoretical model grounded in the collected interview data illustrating adherence behaviour in young adults with asthma was then constructed (Figure 1).

### The Ethical considerations

Ethical approval was granted by the Regional Ethical Review Board at the University of Gothenburg in 2006 (reg. no. 486-06). The participants received both verbal and written information about the aim and utility of the study. They were further told about confidentiality, that their participation was voluntary and that they could withdraw from the study at any time without stating a reason.

## Results

The core category *"A functional day as desired by the person" *was described as having a normal life, which included expectations of being able to manage anything a person without asthma could. Various examples were given, such as physical activities both in terms of exercise and daily activities like climbing the stairs or running for a bus, but also being able to burst into laughter without experiencing symptoms. In brief, an everyday life in which the asthma did not show its face. Most of them reported having achieved a desired functional day. However, it was evident that some of the participants experienced restrictions in relation to certain physical activities, yet they perceived their everyday life with asthma as satisfactory. This perception could be explained by the comparisons they made between today's asthma function and previous function during childhood, when the asthma was experienced as much severer. A functional day seemed to regulate efforts to achieve this outcome.

As illustrated in Figure 1, three types of regulatory foci were identified: Promotive, Preventive and Permissive, which regulated three types of medication tactics: Approaching illness control, Avoiding uncontrolled illness and Acting on the spur of symptoms.

### Promotive focus

This focus was characterized by an eagerness to strive for a positive asthma outcome. This thinking stemmed from a strong wish to live a life equal to that of persons without asthma. Participants associated with this focus aspired to accomplish all of the important things in life without being disturbed by the asthma. Therefore a promotive focus was aimed at progressing with the medication tactic to attain a functional day.

#### Asthma playing in the background

When the asthma was playing in the background, no signs of illness were present in everyday life. The asthma did not interfere with activities in daily life, which allowed the participants to do things that played an important role in their social life. Another advantage of having asthma in control was the possibility to manage other daily activities without experiencing restrictions.

*"It means that I, for example, have the energy to climb the stairs much more easily than I would without medication. Without standing and panting afterwards and feeling the tightness and all. Or when I run to the bus when I'm about to miss it. Things like that." *(4)

### Approaching controlled illness

#### Promotive adherence reasoning

This reasoning was influenced by the aspiration to achieve a functional day. Adhering to the asthma medication prescription was thus viewed as advancement towards this ideal state when the asthma only plays in the background. Some medication worries were put forward, but the benefits seemed to exceed the concerns. It was argued that this medication tactic was considered promising because it was expected to promote a preferred lifestyle.

*"You want to do exercise, for example, because that's important for your body. So you get to take a little medicine so you can manage it. Anyway it's a good reason to take medicine. It's great. So then you take it to feel good." *(7)

Participants who had adopted this type of adherence behaviour had noted that it generated a positive outcome reflected in an asthma day that was easily managed. When they had found themselves between prescriptions, they had experienced episodes during which the desired outcome had not been attained. Such experiences were used as opportunities to compare periods when the asthma was playing in the background with periods when the asthma was showing its face. Eventually, these experiences seemed to function as evidence of the gains associated with their present medication tactic.

*"It's because I know it makes me feel better. I have tested, for example, when I've run out of medicine and not received the new prescription yet. So I've been without it a while and I simply notice the difference in how I feel. So that's why I'm careful with it, because I know I feel better and that everyday life is easier for me. Well, that's the simple reason." *(4)

#### Promotive medication adjustment

This medication tactic was aimed at achieving a desired asthma outcome by using a minimum of asthma medication. Thus, medication use was adjusted depending on estimated need without jeopardizing a positive outcome. One advantage of this strategy was that the body was not exposed to medication unnecessarily, while no clear concerns about side effects were expressed. This tactic rested on the opinion that every person knows best how his/her own body functions - even better than the doctors who made their assessment during a short clinical visit, sometimes without performing medical tests. This thinking led to medication breaks of varying duration. Some had been off medication for almost a year, but were still able achieve a functional day. Others had shorter breaks, for instance during stays abroad for a couple of weeks to several months when the asthma was perceived to function satisfactorily without medication.

*"So I don't really follow the doctor's orders to the letter, instead I have a feeling for what's needed. And if I'm just .... going on a trip for two weeks then I don't need to take it just because I'm travelling." *(14)

Shorter breaks during periods requiring less physical exertion also occurred. It was stressed that this strategy was not recommended for persons newly diagnosed with asthma, because it required experience. It was also expressed that one risk associated with this strategy is that one could unconsciously get used to poorer asthma function.

### Preventive focus

This focus was grounded in a sense of responsibility one had to oneself to be spared from a situation in which the asthma dominated everyday life. Participants associated with this focus tended to act in an anticipatory way to keep the signs of illness away. This thinking was shown in their adherence behaviour, which was motivated by the need to ensure that asthma would not play in the foreground.

#### Asthma playing in the foreground

When the asthma played in the foreground, it showed its face as typical asthma symptoms. These episodes varied in length and severity, but were perceived as a direct consequence of not having taken the asthma medication as usual.

*"Then my voice gets hoarse. It can be a little tough when I wake up, sometimes breast pain when I breath and I feel ... so I lose my voice often and at times it's hard to talk and I'm clearing my voice all the time, that's what happens. I don't feel quite as alert either." *(1)

It was not only the occurrence of symptoms that was experienced as troublesome when the asthma set the agenda, but also its effect on daily life as a whole. Such a period was described as being characterized by poor strength and difficulty managing ordinary physical activities like climbing the stairs. An insufficient ability to stay focused that negatively affected their work or studies was also described.

### Avoiding uncontrolled illness

#### Preventive adherence reasoning

This medication tactic was aimed at avoiding being controlled by the asthma. Simultaneously, it was expected to secure a functional day. Taking the asthma medication as recommended was therefore considered both as a necessary investment and as an essential tactic. Despite a few concerns with intake of asthma medication, this medication tactic was regarded as a precautionary measure that was believed to prevent the asthma from playing in the foreground, both at present and in the future.

*"... I take them because I know I won't feel well if I don't take them. So somewhere in the back of my mind there's something saying I have to take them in order to get through the day." *(15)

*"It may get worse in the future ... if I don't take my medicine now, I probably won't feel good in the future, and I think about that." *(1)

It was also argued that this tactic had spared them from severe asthma attacks in the past. Some participants had experienced troublesome asthma attacks that had etched fearful memories in their minds. These were incidents they never wanted to relive again. Some even expressed worries about not currently having a sufficient medication treatment, despite claiming to experience a functional day. Others knew about other people's asthma mismanagement and concomitant severe attacks. Thus, taken together, these experiences functioned as cautionary examples that influenced their current adherence behaviour.

It was explained that managing asthma through preventive adherence behaviour had an overall advantage in terms of the sense of calm that grows out of being certain one is safe.

*"Well, then it's like you take it to feel ... feel secure and protected. You don't have to feel bad as often." *(11)

#### Preventive medication overuse

Another preventive medication tactic was to overuse the asthma medication. This tactic seemed to be associated with distrust that the current prescription of asthma medication provided comprehensive protection from a negative asthma outcome. Preventive overuse was deliberately put into practice as an additional guarantee that the asthma would have no opportunity to play in the foreground. In these cases, the asthma was considered a very troublesome disease and a watchful eye was constantly kept on possible emerging asthma symptoms. This tactic was manifested by taking both the preventive and reliever asthma medication more times a day than was prescribed.

*"I'm supposed to take my preventer inhaler daily, morning and evening, but sometimes I take it several times a day. Along with my rescue inhaler." *(8)

Yet another type of preventive overuse was described that only involved the reliever medication. It was regarded as a required preventive measure to make sure that the asthma would not play in the foreground, for example when performing strenuous physical activities. The experience of side effects such as tremor and palpitations was thought to be a worthwhile investment given the benefit of this protective medication tactic.

### Permissive focus

This focus was aligned with a kind of "let-it-go" mentality, according to which everything will turn out fine. Some skeptical voices towards intake of asthma medication were raised, but they were not heard in general. Participants associated with this way of thinking gave the expression of being rather indifferent in relation to asthma and its outcome. Despite the fact that they had experienced severe attacks, they acted as if their everyday life was seemingly unaffected by asthma. They vaguely described a desired asthma outcome as being relieved from emerging symptoms.

#### Asthma playing out of earshot

The asthma was explained as something that was included in the participants' daily life and that they did not pay much attention to. One good reason was that asthma was given low priority due to another, more troublesome disease. Another was that the accuracy of the asthma diagnosis was doubted. It was argued that asthma symptoms could just as well be experienced by all people, even those without an asthma diagnosis. There was an obvious paradox in these lines of reasoning, as being completely without asthma medication was not an option.

*"Of course I want the rescue inhaler - I don't want to get rid of that. ... ... but maybe even people in general sometimes have trouble breathing ... I don't know what's normal." *(10)

### Acting on the spur of symptoms

#### Permissive adherence reasoning

This medication tactic was justified by the explanation that the participants' asthma mostly was playing out of earshot. However, when the symptoms became more pronounced, they triggered the intake of asthma medication. Although these participants described their medication tactic as not recommendable, they seemed unmotivated to change tactics. No benefit of this medication tactic was expressed. Instead it was put forward that regular medication behaviour would be more advantageous. Still, they reported not really wanting to change their medication tactics.

*"I don't think I'll change anything, I'll just keep doing what I do now or .... although it would probably be good to develop a routine for this. But I don't think I'll do anything about it." *(13)

#### Unstructured medication use

Participants associated with a permissive focus had no integrated routines for their medication intake. They seldom prepared themselves before contact with known asthma triggers, meaning that they did not act until the asthma was playing within earshot. Consequently, they reported unstructured adherence behaviour, implying that the asthma medication was not taken regularly.

*"No, it feels like it's been a long time since I was good at taking the preventer inhaler so I really don't know. No, I don't know. I'm really bad about taking my medications, that's all." *(10)

Sometimes the medication was taken according to prescription, then it was not taken at all for a few weeks or even longer periods. Still the asthma function was experienced as satisfactory, on the whole.

## Discussion

The present findings demonstrated that adherence behaviour in relation to regular asthma medication treatment was grounded in conscious reasoning strongly influenced by a desire to have *a functional day*. Three foci regulated participants' adherence behaviour, as illustrated in the emergent theoretical model (Figure 1). A *promotive focus *contained an ambition to achieve controlled illness, which inspired two types of medication tactics: *promotive adherence reasoning *and *promotive medication adjustment*. A *preventive focus *was seen as ensuring avoidance of uncontrolled illness and led to *preventive adherence reasoning *and *preventive medication overuse*. A *permissive focus *led to *permissive adherence reasoning *and *unstructured adherence behaviour*, where asthma symptoms triggered medication intake.

Several methods exist for assessing adherence, but in a clinical setting questioning is a common method [9]. There are indications that accurately determining the level of a patient's adherence could be a difficult task in the context of a clinical meeting [36-40]. Looking at the adherence reasoning among the participants, it became clear that they did not speak of adherence as a quantitative measure or a certain adherence level. Instead, their main priority in taking asthma medication was to have *a functional day*. On their way to this desired state, they were guided by the three motivational foci, which led to different medication tactics. This indicates that having a goal for one's adherence behaviour is of greater significance to the patient than adhering or not adhering to a prescribed dosage.

The study shows that all participants had consciously adopted a functioning medication tactic that served their needs. From a medical perspective, the presented adherence behaviour probably leaves much to be desired. To arrive at a "win-win adherence outcome", the gap between a patient and medical adherence perspective needs to be bridged, which requires a mutual effort between the patient and the health-care personnel. Patient-centred care stipulates a cooperative effort intended to bring about the best possible health outcome. Health-care personnel are supposed to respect patients' needs and preferences, but also to empower patients to make informed health choices. Patients' responsibility is to actively participate in care and to strive for a better health outcome, which includes adherence to treatment [41].

With regard to the current findings, identifying what a functional day means to the patient could be a good starting point to jointly find a proper medication tactic and to bridge the gap between the two perspectives.

### Methodological considerations

The reason for conducting this study by using a qualitative method was the nature of the study's aim. The GT methodology offers an inductive strategy for collecting and analyzing qualitative data that ends in a theoretical model grounded in data. This method led the researchers to a novel understanding of adherence behaviour in young adults with asthma by exploring their thought processes and reasoning about their medication intake. The theoretical model explaining adherence behaviour was built through a systematic and an analytic research process and therefore has explanatory power for the sample from which the data was collected. When sampling for a qualitative study the focus is not on the representativeness of the population in the same way as in a quantitative method but on the representativeness and variation of the concepts. In the current study, we focused on the indicatives of adherence behaviour to saturate the categories not on the number of interviewees as one interview can give several aspects of the phenomenon under investigation. The theoretical model was not validated statistically because it according to GT methodology was validated throughout the research process by constant comparison of data [29]. This does not rule out that the model could be tested statistically in future studies in samples designed for statistical methods.

Possible limitations of the current study are that the participants were homogenous as regards age and that they had lived most of their lives with asthma. This homogeneity could also be the strength of the study, as the findings are based on rather long lived experience of having asthma. No data on asthma severity were collected, which could be a weakness because degree of severity may influence adherence behaviour. The emerged theoretical model was grounded in data derived from a specific context in terms of a sample consisting of young adults and compared to a quantitative study based on a small sample size, which in fact is a common procedure in qualitative research. For these reasons, the findings may not be transferable to other groups with asthma, which indeed is an indication of the need for further studies.

A potential strength of the current study is that it can add another previously neglected variable to the concept of phenotypes. By definition, the term "phenotype" includes an organism's behaviour, but in current studies of phenotypes of asthma, behaviour and personality are not attended to. Thus, until now, the term phenotype in relation to asthma has included "observable characteristics of asthma such as physiology, triggers, inflammation and response to medication treatment" [42] but has ignored behaviour. For example, the patient's behaviour, including adherence to prescribed medication, can change the degree of expression of disease and may influence severity of asthma. Therefore, the currently described concept showing that three different foci of different asthmatic individuals regulated different kinds of adherence behaviour, will have vast impact on the expression of disease. All three foci are directed towards one desired goal: *a functional day as desired by the person*. Thus, participants associated with a *promotive focus *were active in achieving a mild phenotype and participants associated with a *preventive focus *were active in avoiding a severe phenotype. In contrast, participants associated with a *permissive focus *seemed more likely to act on the asthma syndrome i.e. the symptoms, allowing for a more severe phenotype at some occasions. Therefore, behavioural aspects should be carefully assessed in describing asthma phenotypes, and future development of questionnaires identifying patients with different foci could help in this regard.

## Conclusions

As all participants had consciously adopted functioning medication tactics that directed them towards the desired goal of a functional day. As an effort to bridge the gap between a patient and a medical adherence perspective, patients need support from health-care personnel in defining their desired functional day and guidance in achieving an appropriate medication tactic.

## Competing interests

The authors declare that they have no competing interests.

## Authors' contributions

MA: Designed the study. Carried out data collection, analysis and interpretation of the data and drafted the manuscript. JLÖ: Participated in the conception and initiation of the study. Critically influenced important intellectual content, and influenced the manuscript content. JLU: Participated in interpretation of data. Critically influenced important intellectual content, and influenced the manuscript content. EB: Participated in designing the study, analysis and interpretation of data. Critically influenced the manuscript content. All authors have given their approval of the version to be published.

## Pre-publication history

The pre-publication history for this paper can be accessed here:

http://www.biomedcentral.com/1471-2458/11/809/prepub
